# Racial differences in CD8^+^ T cell infiltration in breast tumors from Black and White women

**DOI:** 10.1186/s13058-020-01297-4

**Published:** 2020-06-09

**Authors:** Yara Abdou, Kristopher Attwood, Ting-Yuan David Cheng, Song Yao, Elisa V. Bandera, Gary R. Zirpoli, Rochelle Payne Ondracek, Leighton Stein, Wiam Bshara, Thaer Khoury, Christine B. Ambrosone, Angela R. Omilian

**Affiliations:** 1 Department of Medicine, Roswell Park Comprehensive Cancer Center, Buffalo, NY USA; 2 Department of Biostatistics & Bioinformatics, Roswell Park Comprehensive Cancer Center, Buffalo, NY USA; 3 Department of Epidemiology, University of Florida, 2004 Mowry Road, Gainesville, FL 32610 USA; 4 Department of Cancer Prevention and Control, Roswell Park Comprehensive Cancer Center, Buffalo, NY USA; 5 Department of Biostatistics and Epidemiology, Rutgers School of Public Health, Piscataway, NJ USA; 6 Cancer Epidemiology and Health Outcomes, Rutgers Cancer Institute of New Jersey, New Brunswick, NJ USA; 7 Slone Epidemiology Center, Boston University Medical Campus, Boston, MA USA; 8 Department of Pathology, Roswell Park Comprehensive Cancer Center, Buffalo, NY USA

**Keywords:** CD8^+^, Breast cancer, Disparities, Immune infiltrates

## Abstract

**Background:**

African American/Black women with breast cancer have poorer survival than White women, and this disparity persists even after adjusting for non-biological factors. Differences in tumor immune biology have been reported between Black and White women, and the tumor immune milieu could potentially drive racial differences in breast cancer etiology and outcome.

**Methods:**

We examined the association of CD8^+^ cytotoxic T cells with clinical-pathological variables in the Women’s Circle of Health Study (WCHS) population of predominantly Black breast cancer patients. We evaluated 688 invasive breast tumor samples (550 Black, 138 White) using immunohistochemical staining of tissue microarray slides. CD8^+^ T cells were scored for each patient tumor sample with digital image analysis.

**Results:**

Black women had a significantly higher percentage of high-grade, estrogen receptor (ER)-negative, and triple-negative tumors than White women and significantly higher CD8^+^ T cell density (median 87.6/mm^2^ vs. 53.1/mm^2^; *p* < 0.001). Within the overall population and in the population of Black women only, CD8^+^ T cell density was significantly higher in younger patients and patients with high-grade and ER/PR-negative tumors. No significant associations were observed between CD8^+^ T cell density and overall survival or breast cancer-specific survival in the overall population, or when Black patients were analyzed as a separate group. However, when stratified by subtype, Black women with triple-negative breast cancer and high CD8^+^ T cell density showed a trend towards better overall survival in comparison with patients with low CD8^+^ T cell density (HR = 0.51; 95% CI 0.25–1.04).

**Conclusions:**

Our data raise the possibility that distinct mechanisms of immune cell action may occur in different racial groups.

## Background

Breast cancer is the second leading cause of female cancer mortality in the USA [1]. Research and treatment advances have significantly lowered breast cancer mortality rates over the years. However, the decline in mortality in African American/Black women continues to lag behind. Breast cancer death rates are 41% higher in Black women than in European American/White women, with an estimated 33,840 new cases and 6540 deaths occurring in Black women in 2019 [1, 2]. The issue of racial disparities in breast cancer outcome and prognosis is a major health care challenge. The reasons underlying this are multifactorial, including socioeconomic status, access to health care, and screening practices [3–6]. However, many studies have found that disparities persist even after adjustment for non-biological factors, and intrinsic differences in tumor biology have been reported, including a higher incidence of estrogen receptor-negative (ER−) cancer in Black women [7–11]. Moreover, recent studies have shown that African ancestry is associated with more pronounced systemic inflammatory responses than European ancestry [12–14], indicating that differences in the tumor immune milieu are also likely to exist, and this could potentially drive racial differences in breast cancer etiology and outcome.

Tumor immune response is a complex phenomenon involving multiple immune cell populations. Tumor-infiltrating lymphocytes (TILs) represent a heterogeneous population of T cells, B cells, and natural killer cells, among others, and are commonly considered the manifestation of the host antitumor immune response. T lymphocytes, particularly cytotoxic CD8^+^ T cells (CTLs), play a crucial role in cell-mediated antitumor immunity by targeting tumor antigen peptides for eradication [15]. Activated CD8^+^ T cells have a potent functional capacity, including degranulation and production of IFN-γ, which is known for its induction of tumor apoptosis and inhibition of angiogenesis [15]. CD8^+^ T cells have been investigated as a biomarker of the tumor immune response in many studies [16–18].

The infiltration of CTLs into the tumor microenvironment is mostly associated with a favorable prognosis in various types of solid malignancies including breast cancer, particularly the HER2-positive and triple-negative (TNBC) subtypes [16, 18]. CTLs also have been shown to predict pathological complete response to primary systemic therapy in breast cancer [19, 20] and the spatial location of CTLs has been shown to influence response to checkpoint blockade in melanoma [21]. However, many studies have shown that even when antitumor TILs such as CTLs are present, tumors continue to progress, suggesting that there are multiple negative immunoregulatory mechanisms that hinder the T cell-mediated antitumor response in the tumor microenvironment (TME) or other forms of immune escape [22, 23].

To date, there is a small but growing body of literature regarding racial differences in tumor immune responses in breast cancer [11, 24–27]. Whereas numerous prior studies have studied the role of immune cells in response to treatment and outcomes in breast cancer, the majority of these studies were conducted in populations of predominantly White women [16, 18]. Characterizations of immune cell populations are lacking in breast tumors from Black women. To address this research gap, we employed immunohistochemical (IHC) staining and quantitative digital imaging to evaluate CD8^+^ lymphocytes in breast tumor samples from patients enrolled in the Women’s Circle of Health Study (WCHS), a case-control study of breast cancer in Black and White women. Our purpose was to characterize and compare the density of CD8^+^ T cells and evaluate their prognostic value in Black women with breast cancer.

## Methods

### Study population

The WCHS is a multi-site, case-control study designed to evaluate the risk factors for aggressive breast cancer in Black and White women. Details on the study recruitment have been described elsewhere [28]. Briefly, participants were 20–75 years old; self-identified as Black or White; had primary, histologically confirmed invasive breast cancer or ductal carcinoma in situ (DCIS); and had no previous history of cancer other than non-melanoma skin cancer. Cases were first identified from several hospitals in New York City and then from several counties in New Jersey using rapid case ascertainment by the New Jersey State Cancer Registry (NJSCR). As part of the informed consent process, patients were asked to sign a release permitting the use of their tumor tissue blocks for research, and then tumor tissues and pathology reports were requested from treating hospitals. Clinicopathologic variables were extracted from the pathology reports: tumor size, grade, lymph node status, molecular subtype (ER, PR, and HER2 status), and whether the patient received neoadjuvant therapy. We obtained neoadjuvant chemotherapy information from the staging notation and a text search function on pathology reports. Breast cancer subtypes were inferred from ER, PR, and HER2 status information on the pathology reports and were as follows: luminal A (ER+ and/or PR+/HER2−), HER2-positive (ER+ and/or PR+/HER2+ or ER− and/or PR−/HER2+), and triple-negative (ER−/PR−/HER2−).

In this study, we used previously built tissue microarrays (TMAs) that consisted of cases only. This included 734 incident, primary invasive breast cancer cases diagnosed from 2001 to 2017. Forty-six patients who received neoadjuvant therapy were excluded, leaving 688 cases (550 Black and 138 White). The number of Black cases greatly exceeds the number of Whites, and this is due to the study design for WCHS, which focused on recruiting Black women. This study was approved by the Institutional Review Boards at Rutgers Cancer Institute of New Jersey and Roswell Park Comprehensive Cancer Center.

### TMAs and immunohistochemistry

TMAs consisted of formalin-fixed paraffin-embedded (FFPE) tumor cores that were selected by a board-certified breast pathologist (TK) based on a review of hematoxylin and eosin (H&E)-stained slides. TMA cores ranged in size from 0.6 to 1.2 mm in diameter, and most patient tumors were represented by at least two TMA cores (range 1 to 6 cores). IHC assays were performed by the Pathology Network Shared Resource (PNSR) at Roswell Park. Briefly, FFPE TMA sections were cut at 4 μm, placed on charged slides, and dried at 60 °C for 1 h. The slides were cooled to room temperature, placed on the Dako Omnis autostainer, and stained with the accompanying Dako antibody and secondary reagents following the manufacturer’s recommendations. TRS High was used for epitope retrieval for 30 min, and the CD8 antibody (clone CD8/144B) was applied at 1/50 dilution. DAB (Diaminobenzidine) was applied for marker visualization. Tonsil tissue was stained in parallel as a positive control, and a negative staining control was performed by applying all staining reagents except for the primary antibody to tonsil tissue. A small number of whole sections (*N* = 10) that matched to cases included in the TMAs were stained using the same methods as a check for using cases in TMA format. To reduce staining variability that can occur with immunohistochemistry, we used an automated staining platform with temperature and humidity controls and clinical-grade reagents, and stained all TMAs in a single batch.

### CD8^+^ T cell digital quantification

TMA slides were digitally scanned using Aperio ScanScope (Leica Biosystems Inc., Buffalo Grove, IL) with a × 20 bright-field microscope and then viewed using Spectrum, the accompanying web-based digital pathology system. Aperio ImageScope version 12.3.3.7014 was used to view images, create annotation layers, and circle areas of interest for analysis. The Aperio software was used to develop quantitative image analysis algorithm macros to score the IHC slides. Briefly, these algorithms used color de-convolution to distinguish DAB from the hematoxylin counterstain and provide stain separation.

Although CD8 stains the membrane compartment, the low cytoplasmic volume of T cells causes the stain to appear to cover nearly all of the cell area. Because of this, the cytoplasmic algorithm in Aperio was shown to perform better at quantifying the number of positively stained T cells; this was visually confirmed by a pathologist (WB). The cytoplasmic algorithm was fine-tuned to detect cell features and was uniformly applied to all TMA cores after our optimization and validation process. Our study pathologist visually inspected a subset of cores, especially when digital counts were high, to ensure that the high counts were reflective of the staining on the slide. Aperio reported the total number of analyzed cells, the percentage of CD8^+^ cells, and the size of the tissue area that was analyzed. In order to normalize the variable results that were produced due to the difference in tissue core sizes, the number of CD8^+^ lymphocytes in each patient sample was reported per square millimeter of tumor tissue. Nine cores lacking sufficient tumor cellularity (< 50 tumor nuclei per core) were excluded from our analysis [29]. The average CD8^+^ cell density across multiple cores from each patient was used for analyses.

As many studies of tumor-infiltrating lymphocytes report on the stromal compartment, we circled the tumor areas and stromal areas in a subset of cases (*N* = 96) with the intention of determining if CD8 expression differs between the two regions. There was no significant difference in T cell densities between the two compartments (sign test, *p* = 0.337), so we did not continue manual circling because it is extremely labor-intensive.

### Outcome variables

Data on vital status, including dates and causes of death, were available from cases who were enrolled in New Jersey through linkage with the New Jersey State Cancer Registry files. The ICD-10 code (C50) was used to identify breast cancer mortality. Follow-up time was calculated from the date of enrollment to the last contact date (censored) or date of death. Survival outcomes were available for 590 women (488 Black and 102 White), including 85 events, as of October 31, 2018. The mean follow-up time was 5.75 years (interquartile range = 3.92–9.17 years).

### Statistical analysis

Patient demographic and clinical characteristics were summarized overall and by race using the median and interquartile range (IQR) for continuous variables, and frequencies and relative frequencies for categorical variables. CD8^+^ T cell density was evaluated in invasive primary breast tissue for 688 unique patients (*N* = 550 Black, *N* = 138 White). Separate general linear models were used to model CD8^+^ T cell density as a function of race, each clinicopathological characteristic, and their two-way interaction. *F* tests about the appropriate contrasts of model estimates were used to (a) evaluate, within race, the association between CD8^+^ T cell density and each characteristic and (b) compare racial groups within each level of a characteristic. For the race comparisons, Tukey’s adjustment was applied. Model assumptions were verified graphically, and a log-transformation was applied to CD8^+^ T cell density. ANCOVA analysis was conducted on the association between race and CD8^+^ T cell density, adjusting for grade, and breast cancer subtype, which were associated with both race and CD8 expression.

Overall survival (OS), defined as the time from diagnosis to the date of the last contact or of death from any cause, and breast cancer-specific survival (DSS), defined as the time from diagnosis to the date of the last contact or death from breast cancer, were summarized by race and categorized CD8^+^ T cell density using Kaplan-Meier methods and compared using the log-rank test. Estimates of the median and 5/10-year survival rates were obtained with 95% confidence intervals. As there are currently no established cutoff points, CD8^+^ T cell density was dichotomized at the cohort-specific median into low and high expression. Multivariable Cox regression models were used to evaluate the association between CD8^+^ T cell density and survival outcomes while adjusting for age. Additional models were explored that adjusted for grade, race, and subtype, and we also examined different cutoff points for categorizing CD8^+^ T cell density as high vs. low. All analyses were conducted in SAS v9.4 (Cary, NC) with a two-sided significance level of 0.05.

## Results

The demographic and clinicopathological characteristics of the study population are shown in Table 1. Consistent with other studies, Black women in the WCHS had a significantly higher percentage of high-grade, ER-negative, and triple-negative tumors than White women. There was a difference in OS, with White women having better outcomes, although it was borderline significant (HR = 0.58; 95% CI 0.33–1.03; *p* = 0.06). The DSS followed the same trend in this population (HR = 0.54; 95% CI 0.26–1.13; *p* = 0.10).

**Table 1:** Patient demographic and primary invasive tumor characteristics among cases in WCHS, 2001–2017

		Black	White	Overall
Overall	*N*	550 (79.9%)	138 (20.1%)	688 (100%)
Age at diagnosis	Median/IQR	55.0/(46, 62)	52.5/(45, 60)	54.5/(46, 61)
Subtype	Luminal A	322 (59.3%)	91 (65.9%)	413 (60.6%)
	HER2-positive	80 (14.7%)	28 (20.3%)	108 (15.9%)
	Triple-negative	141 (26.0%)	19 (13.8%)	160 (23.5%)
ER	Positive	366 (66.7%)	107 (77.5%)	473 (68.9%)
	Negative	183 (33.3%)	31 (22.5%)	214 (31.1%)
PR	Positive	284 (52.1%)	82 (59.4%)	366 (53.6%)
	Negative	261 (47.9%)	56 (40.6%)	317 (46.4%)
HER2	Positive	80 (14.7%)	28 (20.3%)	108 (15.8%)
	Negative	464 (85.3%)	110 (79.7%)	574 (84.2%)
Stage	I	221 (40.2%)	62 (45.3%)	283 (41.2%)
	II	250 (45.5%)	57 (41.6%)	307 (44.7%)
	III/IV	79 (14.4%)	18 (13.1%)	97 (14.1%)
Grade	Low	69 (12.6%)	29 (21.5%)	98 (14.4%)
	Intermediate	186 (34.1%)	60 (44.4%)	246 (36.1%)
	High	291 (53.3%)	46 (34.1%)	337 (49.5%)
Tumor size	< 1.0	54 (9.9%)	21 (15.3%)	75 (11.0%)
	1–1.9	199 (36.4%)	54 (39.4%)	253 (37.0%)
	≥ 2.0	294 (53.7%)	62 (45.3%)	356 (52.0%)
LN status	Positive	221 (41.2%)	51 (37.5%)	272 (40.4%)
	Negative	316 (58.8%)	85 (62.5%)	401 (59.6%)
OS	10-year rate	0.78	0.87	0.81
	95% CI	0.72–0.84	0.79–0.92	0.76–0.85
DSS	10-year rate	0.84	0.92	0.87
	95% CI	0.78–0.89	0.84–0.96	0.82–0.90

*ER* estrogen receptor, *PR* progesterone receptor, *HER2* human epidermal growth factor receptor 2. Four staging categories, I–IV, from the American Joint Committee on Cancer (AJCC) were examined; stage 0 patients were not included in this study. Low tumor grade denotes well-differentiated tumors, intermediate denotes moderately differentiated, and high grade denotes poorly differentiated tumors. Tumor size (cm) was classified into three categories: < 1.0 cm, 1.0–2.0 cm, and > 2.0 cm. Lymph node (LN) status was defined as the presence (positive) or no (negative) cancer cells in the axillary lymph nodes

### CD8^+^ T cells in Black and White women

**Fig. 1:**
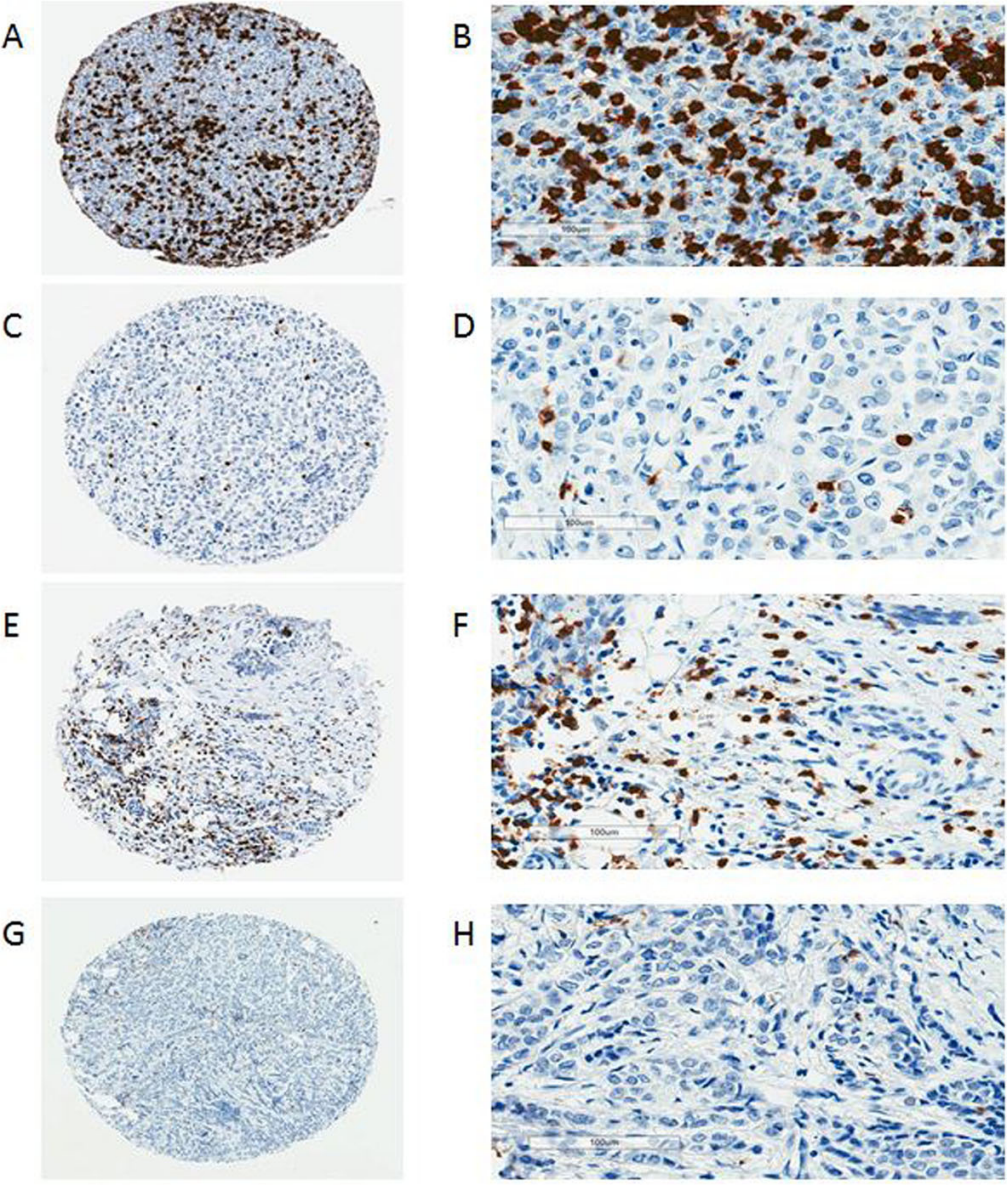
Representative CD8 immunohistochemical staining in breast tissue microarray cores. **a** Black participant with high CD8^+^ T cell density, **b** magnified. **c** Black participant with low CD8^+^ T cell density, **d** magnified. **e** White participant with high CD8^+^ T cell density, **f** magnified. **g** White participant with low CD8^+^ T cell density, **h** magnified

Representative staining of TMA cores is shown in Fig. 1. Black patients had a significantly higher CD8^+^ T cell density compared to White patients (*p* < 0.001), with median values of 87.6/mm^2^ and 53.1/mm^2^, respectively (Table 2). Higher CD8^+^ T cell density in tumors from Black patients was observed for all three breast cancer subtypes. The highest CD8^+^ T cell density was in triple-negative tumors from Black patients, whereas the lowest occurred in luminal tumors from White women (Table 2). Significantly higher CD8^+^ T cell density in tumors from Black patients remained after adjusting for tumor grade and subtype with ANCOVA (mean difference = 156.5/mm^2^; 95% CI 31.1–281.9/mm^2^; *p* = 0.002).

**Table 2:** CD8^+^ T cell expression (per mm^2^) and patient factor interactions with race. The associations between CD8^+^ T cell density and clinicopathological characteristics were evaluated and stratified by race using general linear models. Racial comparisons within each factor level used Tukey-adjusted pairwise comparisons

		Blacks			Whites			Interaction *p* value	B vs. W within level
		*n*	Median (IQR)	*p* value	*n*	Median (IQR)	*p* value		
Total		550	87.6 (24.9, 271.7)		138	53.1 (14.1, 131.4)			< 0.001
Age	< 40	59	163.6 (34.7, 742.0)	0.005	12	82.5 (16.4, 240.5)	0.58	0.93	0.81
	40–49	134	95.5 (28.6, 252.9)		42	59.3 (24.6, 152.6)			0.59
	50–59	184	104.3 (27.5, 279.6)		48	45.4 (17.6, 106.3)			0.13
	60+	173	62.4 (16.5, 196.6)		36	47.5 (11.8, 119.9)			0.70
Subtype	Luminal A	322	66.6 (22.4, 169.6)	< 0.001	91	49.0 (13.2, 105.1)	0.56	0.43	0.30
	HER2-positive	80	115.2 (33.7, 334.2)		28	69.5 (18.6, 149.2)			0.55
	Triple-negative	141	183.1 (33.2, 838.1)		19	56.7 (12.9, 269.3)			0.14
ER	Positive	366	70.7 (23.5, 184.4)	< 0.001	107	49.0 (13.7, 130.3)	0.82	0.12	0.12
	Negative	183	155.3 (30.3, 694.5)		31	60.9 (16.4, 152.6)			0.01
PR	Positive	284	68.2 (24.8, 169.3)	< 0.001	82	53.1 (19.3, 121.3)	0.62	0.05	0.43
	Negative	261	135.4 (26.9, 491.3)		56	54.7 (12.1, 157.4)			< 0.001
HER2	Positive	80	115.2 (33.7, 334.2)	0.20	28	69.5 (18.6, 149.2)	0.41	0.99	0.35
	Negative	464	83.7 (24.3, 268.1)		110	50.5 (13.2, 131.4)			0.003
Stage	I	221	101.2 (27.4, 246.5)	0.97	62	34.8 (9.8, 83.0)	0.01	0.04	< 0.001
	II	250	79.9 (22.9, 295.8)		57	66.1 (23.8, 166.0)			0.91
	III/IV	79	99.7 (24.4, 296.1)		18	68.4 (37.5, 226.3)			1.00
Grade	Low	69	45.2 (24.6, 131.0)	< 0.001	29	38.5 (9.1, 76.6)	0.13	0.82	0.60
	Intermediate	186	71.9 (23.0, 162.6)		60	45.8 (13.7, 122.2)			0.63
	High	291	138.8 (27.1, 514.6)		46	78.6 (24.6, 170.9)			0.22
Tumor size	< 1.0	54	68.8 (22.6, 235.0)	0.30	21	60.7 (11.0, 88.4)	0.20	0.19	0.83
	1–1.9	199	105.2 (30.3, 321.5)		54	45.8 (11.3, 121.3)			0.004
	≥ 2.0	294	84.7 (21.6, 274.8)		62	58.4 (20.7, 152.6)			0.79
LN status	Positive	221	85.6 (23.4, 335.5)	0.78	51	60.9 (18.8, 152.6)	0.43	0.59	0.22
	Negative	316	90.8 (25.5, 254.1)		85	49.0 (12.9, 123.2)			0.005

### Association of CD8^+^ T cell density with clinicopathologic characteristics

In the overall population and Black population subset, CD8^+^ T cell density was noted to be significantly higher in younger patients, tumors with high grade, and tumors that were negative for ER and PR or triple-negative (Table 2, Supplemental Table 1). There was a significant interaction in which the relationship between CD8^+^ T cell density and disease stage depended on race (*p* = 0.04); White patients had significantly lower CD8^+^ expression in stage I breast cancer compared to stages II and III/IV. There was no relationship between stage and CD8^+^ T cells in the Black population (Table 2).

### CD8^+^ T cell density and survival outcomes

**Fig. 2:**
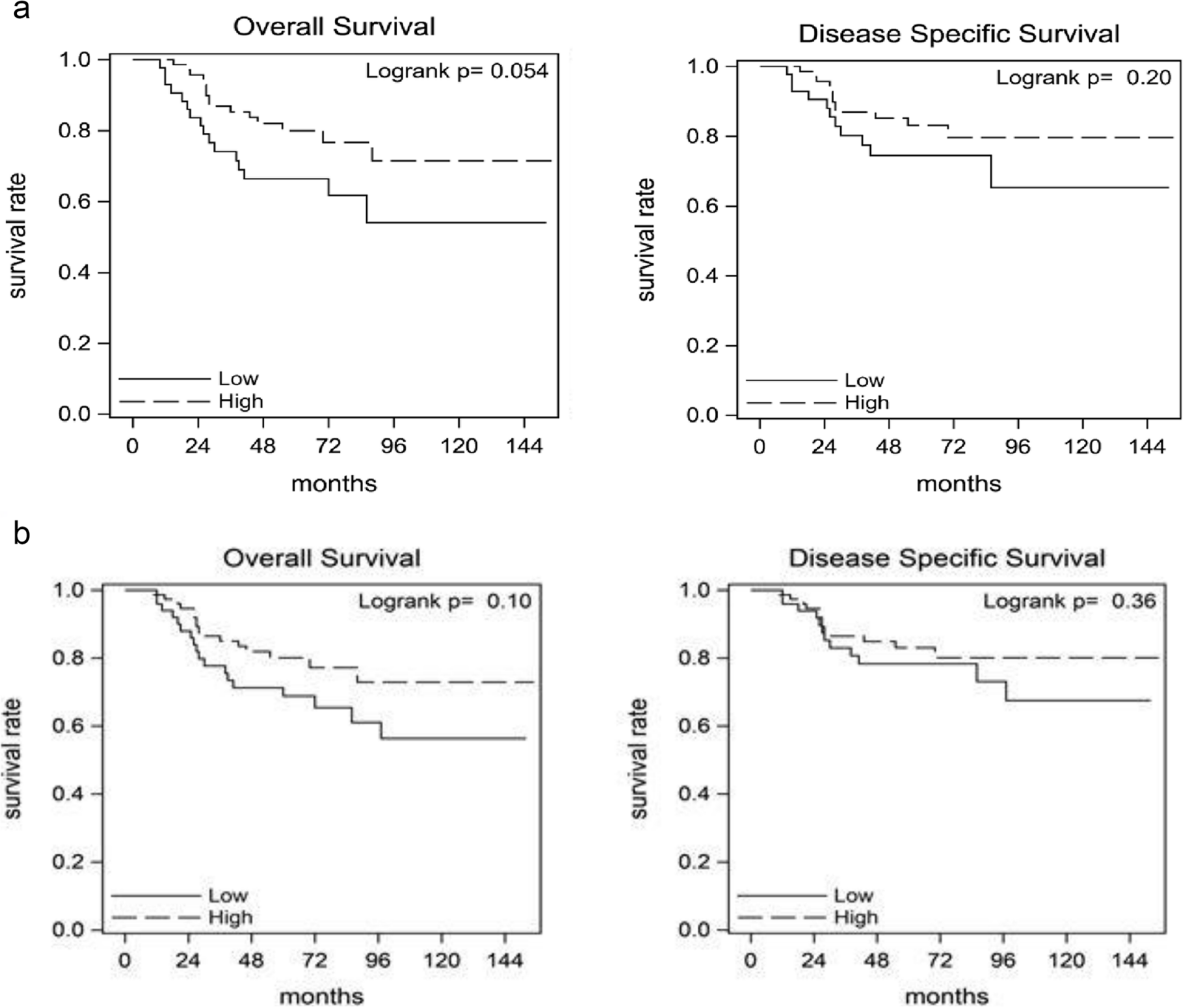
Kaplan-Meier plots of triple-negative breast cancer-specific survival in the WCHS population, stratified by CD8^+^ T cell density. **a** Survival curves for Black patients with triple-negative breast cancer. CD8^+^ T cell density was categorized into low vs. high based on the median value of CD8^+^ cells/mm^2^ for the Black population. **b** Survival curves for Black and White patients with triple-negative breast cancer. CD8^+^ T cell density was categorized into low vs. high based on the median value of CD8^+^ cells/mm^2^ for the overall WCHS population

No significant associations were observed between CD8^+^ T cell density and survival outcomes in the overall population or in the Black population. However, when stratified by breast cancer subtype, we found a trend towards improved OS (HR = 0.51; 95% CI 0.25–1.03) in the Black triple-negative breast cancer patients with high CD8^+^ T cell density compared to those with low CD8^+^ density (Fig. 2a). A similar trend is seen in the overall population (HR = 0.58; 95% CI 0.29–1.13) with triple-negative breast cancer (Fig. 2b). Multivariable analysis that adjusted for age showed a trend towards improved OS in the triple-negative subset of Black patients with high CD8^+^ T cell density in comparison with patients with low CD8^+^ T cell density (HR = 0.51; 95% CI 0.25–1.04; Table 3). Additional models that also adjusted for disease grade or modified the cut-point for high/low expression did not significantly change the hazard ratio or *p* value associated with CD8^+^ T cell density. Survival analyses conducted for each tissue compartment separately (epithelial vs. stromal) were concordant with the results in which the compartments were combined (Supplemental Table 2).

**Table 3:** Age-adjusted associations with survival in Black cases in WCHS

Subtype	CD8	Overall survival		Disease-specific survival	
		Events/*N*	Hazard ratio (95% CI)*	Events/*N*	Hazard ratio (95% CI)*
Luminal A	High vs. low	26/298	1.02 (0.47, 2.24)	15/298	0.66 (0.23, 1.94)
HER2-positive	High vs. low	11/71	1.36 (0.40, 4.63)	9/71	2.48 (0.55, 11.27)
Triple-negative	High vs. low	31/114	0.51 (0.25, 1.04)	23/114	0.58 (0.26, 1.33)

*Hazard ratios are age-adjusted

## Discussion

Immunotherapy is an active and promising area of clinical oncology that has transformed patient care in some malignancies. As a result, the characterization of immune cells in the breast tumor microenvironment has received a lot of attention. Many studies have documented tumor-infiltrating lymphocytes with particular focus on CD8^+^ T cells in populations of White women, and several cohorts of Asian women have also been evaluated [16, 18, 19, 30]. Despite these efforts, very little is known about CD8^+^ tumor-infiltrating lymphocytes in Black women with breast cancer. Black patients are underrepresented in immunotherapy trials, and their unique tumor biology is mostly unaccounted for, adding to the challenge of investigating the efficacy of immunotherapeutic agents in this population [31]. In the few studies that have examined immune infiltrates in Black women with breast cancer, sample sizes were limited [11, 24–27, 32]. Our study is the first to correlate CD8^+^ T cell densities with tumor characteristics and clinical outcomes in a relatively large population of Black women with breast cancer.

In this study, we show that tumor-infiltrating CD8^+^ T cell density is significantly higher in Black breast cancer patients compared to Whites, independent of tumor subtype or grade. Our results add to an emerging trend of studies of the breast tumor microenvironment that indicates Blacks may have different tumor immune milieus than Whites. Black women have higher levels of inflammatory biomarkers and cytokines associated with breast cancer risk and survival [33–38], and genetic variants of cytokine-related genes of the adaptive immune response are associated with higher breast cancer risk in Black patients [39]. In genome-wide DNA methylation studies in tumors from Black and White women, the majority of differentially methylated loci by race in ER− breast cancer were related to immune signaling and immune-inflammatory pathways [40, 41]. Other reports have shown breast tumors from Black women have more prominent interferon signatures, macrophages, and higher immune dysfunction scores [11, 24–27]. A recent integrative analysis of multiple data sources from TCGA showed that breast tumor leukocyte fraction and lymphocytes were higher in women of African descent [32].

Many studies report that the effect of tumor-infiltrating CD8^+^ lymphocytes on the prognosis of breast cancer patients is favorable [16], with triple-negative and HER2-positive showing the highest benefit [18]. In this study, we observe a trend towards improved outcomes in triple-negative breast cancer patients with high CD8^+^ T cell density, although this was not statistically significant. Furthermore, no significant survival associations were otherwise noted with CD8^+^ T cell density in other subgroups. Since most previous work on this topic in breast cancer has been overwhelmingly focused on White or Asian patients, our data raise the possibility that distinct mechanisms of immune cell action may occur in women of African descent.

Alternatively, the lack of a significant association of CD8^+^ T cell density with survival in our predominantly Black population may be due to the sample size and a low number of events in the breast subtypes. Other explanations include that proximity to other immune cells may influence how CD8^+^ cells function in the TME, and this in turn could influence the relationship with survival in Black women. For example, CD8^+^ T cells may be inhibited by macrophages that may be present in higher numbers in breast tumors from Black women [25, 26], or co-occurrence with regulatory cells that we did not examine, but have been associated with poor prognosis in breast cancer [42]. Similarly, the spatial location of tissue-resident memory CD8^+^ T cells expressing CD103^+^ or the proportion of bystander CD8^+^ T cells may be factors [43–45]. However, our current methods preclude us from investigating cell subsets that require multiple markers to determine. Future studies will employ multispectral staining and functional analyses to more comprehensively characterize how immune subsets differ and their spatial relationships in these two racial groups.

Our findings might also be explained by immunoediting in which there is a dynamic interplay between tumor and immune cells that leads to the eventual escape of tumor cells from the surveillance from immune cells. Studies have shown that African ancestry is associated with a stronger response to immune activation [12, 13, 46]. Yet, Blacks fare worse than Whites for many cancers [2], contrary to a wealth of studies that show a strong presence of immune cells in the TME is correlated with better pCR, prognosis, and survival [16–18]. This apparent paradox may be reconciled with the recognition that the immune system works both to constrain and promote tumor development wherein immune cells interact with the host tumor to shape tumor immunogenicity, and tumor subclones may evolve to be more evasive or immunosuppressive [47]. Because women of African descent have stronger immune responses, their own immune systems may select for tumor cells that have resistance to the surrounding immune effector cells. This could lead to higher densities of CD8^+^ T cells in breast tumors from Black women without the concomitant improved outcomes that are typically associated with higher levels of T cell infiltration.

The strengths of our study include the large population of Black women with breast tumor tissues available in the WCHS. We also used quantitative digital analysis of our stained tissues that served to eliminate pathologist subjectivity in scoring. Despite the advantage that TMAs provide in reducing staining variability and allowing us to screen hundreds of cases at once, the use of TMAs rather than whole sections precludes spatial analyses that could offer additional information, such as the prevalence of CD8^+^ T cells at the invasive margins of the tumor. It is possible that lymphocyte location rather than density may be a more prognostic marker for Black patients. However, a whole section approach is cost-prohibitive when staining hundreds of samples. Lastly, low statistical power and number of events limited our conclusions.

## Conclusions

In summary, immune cell infiltrates interact with the host tumor to affect the course of the disease and may play a pivotal role in breast cancer disparities between Black and White women. A thorough evaluation of the immune landscape in breast tumors in different racial groups will inform our understanding of immune escape mechanisms, and how these may differ by ancestry, potentially providing insight for treatment strategies for Black women. Evaluating cytotoxic T cells in a large cohort of Black women is a necessary first step towards these goals. Here, we show that Blacks have significantly higher CD8^+^ T cell density than Whites, but this does not confer a survival advantage. Additionally, within the Black population, there was a trend towards a survival advantage with higher CD8^+^ T cell density only in the triple-negative subgroup. These are intriguing findings that require replication in additional populations of Black women with breast cancer. Future studies are needed to determine the functional properties of CD8^+^ cells in Black women and to characterize additional immune cell subtypes that may also play a role.

## Supplementary information

**Additional file 1: Supplemental Table 1.** CD8^+^ T cell density in breast tumors according to clinicopathological characteristics in Black and White cases in the WCHS, 2001-2017. The associations between CD8^+^ T cell density and clinicopathological characteristics were evaluated in the overall cohort and stratified by race using general linear models. **Supplemental Table 2.** Multivariable associations with survival for each tissue compartment (epithelial vs. stromal).

## Data Availability

The datasets and IHC images analyzed for the current study are available from the corresponding author upon request.

## Abbreviations

CTLs: Cytotoxic CD8^+^ T cells
DCIS: Ductal carcinoma in situ
HR: Hazard ratio
IHC: Immunohistochemistry
NJSCR: New Jersey State Cancer Registry
TILs: Tumor-infiltrating lymphocytes
TME: Tumor microenvironment
WCHS: Women’s Circle of Health Study
