# Supplementary material for: Racial differences in CD8+ T cell infiltration in breast tumors from Black and White women
Source: Breast Cancer Res. 2020 Jun 9;22:62. doi: 10.1186/s13058-020-01297-4 (PMC7285742; doi:10.1186/s13058-020-01297-4)
Supplement: Supplementary file 1 — Additional file 1: Supplemental Table 1. CD8+ T cell density in breast tumors according to clinicopathological characteristics in Black and White cases in the WCHS, 2001-2017. The associations between CD8+ T cell density and clinicopathological characteristics were evaluated in the overall cohort and stratified by race using general linear models. Supplemental Table 2. Multivariable associations with survival for each tissue compartment (epithelial vs. stromal). [file 13058_2020_1297_MOESM1_ESM.docx]

**Supplemental Table 1.** CD8^+^ T cell density in breast tumors according to clinicopathological characteristics in Black and White cases in the WCHS, 2001-2017. The associations between CD8^+^ T cell density and clinicopathological characteristics were evaluated in the overall cohort and stratified by race using general linear models.

|  | All women | | | |  | | Black women | | | |  | | White women | | | |  |
| --- | --- | --- | --- | --- | --- | --- | --- | --- | --- | --- | --- | --- | --- | --- | --- | --- | --- |
|  | | N | Median CD8/mm^2^ | P-value | |  | | N | Median CD8/mm^2^ | P-value | |  | | N | Median CD8/mm^2^ | P-value | |
|  | |  |  |  | |  | |  |  |  | |  | |  |  |  | |
| **Total** | | 688 | 75.7 | - | |  | | 550 | 87.6 | - | |  | | 138 | 53.1 | - | |
|  | |  |  |  | |  | |  |  |  | |  | |  |  |  | |
| **Age (yrs)** | |  |  | 0.004 | |  | |  |  | 0.005 | |  | |  |  | 0.58 | |
| <40 | | 71 | 124.7 |  | |  | | 59 | 163.6 |  | |  | | 12 | 82.5 |  | |
| 40-49 | | 176 | 80.6 |  | |  | | 134 | 95.5 |  | |  | | 42 | 59.3 |  | |
| 50-59 | | 232 | 85.4 |  | |  | | 184 | 104.3 |  | |  | | 48 | 45.4 |  | |
| 60+ | | 209 | 57.3 |  | |  | | 173 | 62.4 |  | |  | | 36 | 47.5 |  | |
|  | |  |  |  | |  | |  |  |  | |  | |  |  |  | |
| **Subtype** | |  |  | <0.0001 | |  | |  |  | <0.001 | |  | |  |  | 0.56 | |
| Luminal A | | 413 | 60.7 |  | |  | | 322 | 66.6 |  | |  | | 91 | 49.0 |  | |
| HER2positive | | 108 | 99.7 |  | |  | | 80 | 115.2 |  | |  | | 28 | 69.5 |  | |
| Triple negative | | 160 | 162.9 |  | |  | | 141 | 183.1 |  | |  | | 19 | 56.7 |  | |
|  | |  |  |  | |  | |  |  |  | |  | |  |  |  | |
| **ER status** | |  |  | <0.0001 | |  | |  |  | <0.001 | |  | |  |  | 0.82 | |
| Positive | | 473 | 65.3 |  | |  | | 366 | 70.7 |  | |  | | 107 | 49.0 |  | |
| Negative | | 214 | 129.7 |  | |  | | 183 | 155.3 |  | |  | | 31 | 60.9 |  | |
|  | |  |  |  | |  | |  |  |  | |  | |  |  |  | |
| **PR status** | |  |  | 0.007 | |  | |  |  | <0.001 | |  | |  |  | 0.62 | |
| Positive | | 366 | 64.3 |  | |  | | 284 | 68.2 |  | |  | | 82 | 53.1 |  | |
| Negative | | 317 | 111.7 |  | |  | | 261 | 135.4 |  | |  | | 56 | 54.7 |  | |
|  | |  |  |  | |  | |  |  |  | |  | |  |  |  | |
| **HER2 status** | |  |  | 0.323 | |  | |  |  | 0.20 | |  | |  |  | 0.41 | |
| Positive | | 108 | 99.7 |  | |  | | 80 | 115.2 |  | |  | | 28 | 69.5 |  | |
| Negative | | 574 | 71.5 |  | |  | | 464 | 83.7 |  | |  | | 110 | 50.5 |  | |
|  | |  |  |  | |  | |  |  |  | |  | |  |  |  | |
| **AJCC Stage** | |  |  | 0.458 | |  | |  |  | 0.97 | |  | |  |  | 0.01 | |
| I | | 283 | 72.0 |  | |  | | 221 | 101.2 |  | |  | | 62 | 34.8 |  | |
| II | | 307 | 79.3 |  | |  | | 250 | 79.9 |  | |  | | 57 | 66.1 |  | |
| III/IV | | 97 | 80.7 |  | |  | | 79 | 99.7 |  | |  | | 18 | 68.4 |  | |
|  | |  |  |  | |  | |  |  |  | |  | |  |  |  | |
| **Grade** | |  |  | <0.0001 | |  | |  |  | <0.001 | |  | |  |  | 0.13 | |
| Low | | 98 | 40.9 |  | |  | | 69 | 45.2 |  | |  | | 29 | 38.5 |  | |
| Intermediate | | 246 | 66.1 |  | |  | | 186 | 71.9 |  | |  | | 60 | 45.8 |  | |
| High | | 337 | 128.0 |  | |  | | 291 | 138.8 |  | |  | | 46 | 78.6 |  | |
|  | |  |  |  | |  | |  |  |  | |  | |  |  |  | |
| **Tumor Size** | |  |  | 0.262 | |  | |  |  | 0.30 | |  | |  |  | 0.20 | |
| <1.0 cm | | 75 | 66.2 |  | |  | | 54 | 68.8 |  | |  | | 21 | 60.7 |  | |
| 1 - 1.9 | | 253 | 85.3 |  | |  | | 199 | 105.2 |  | |  | | 54 | 45.8 |  | |
| >2.0 | | 356 | 75.7 |  | |  | | 294 | 84.7 |  | |  | | 62 | 58.4 |  | |
|  | |  |  |  | |  | |  |  |  | |  | |  |  |  | |
| **LN Status** | |  |  | 0.490 | |  | |  |  | 0.78 | |  | |  |  | 0.43 | |
| Positive | | 272 | 80.6 |  | |  | | 221 | 85.6 |  | |  | | 51 | 60.9 |  | |
| Negative | | 401 | 72.0 |  | |  | | 316 | 90.8 |  | |  | | 85 | 49.0 |  | |

ER = estrogen receptor, PR = progesterone receptor, HER2 = human epidermal growth factor receptor 2. Four staging categories, I-IV, from the American Joint Committee on Cancer (AJCC) were examined, stage 0 patients were not included this study. Low tumor grade denotes well differentiated tumors, Intermediate denotes moderately differentiated, and high grade for poorly differentiated tumors. Tumor size (cm) was classified into three categories: <1.0 cm, 1.0-2.0 cm, and >2.0 cm. LN = Lymph node status and was defined as the presence (positive) or no (negative) cancer cells in axillary lymph nodes.

**Supplemental Tables 2. Multivariable associations with survival for each tissue compartment (epithelial vs. stromal).**

| Stromal CD8 Expression |
| --- |

| **Outcome** | **Variable** |  | **HR (95% CI)** | **P-value** |
| --- | --- | --- | --- | --- |
| OS | Age | 1 Year Increase | 1.01 (0.96, 1.07) | 0.648 |
|  | CD8 | High vs Low | 0.55 (0.18, 1.71) | 0.303 |
|  | Race | European American vs African American | 1.08 (0.39, 3.00) | 0.888 |
| DSS | Age | 1 Year Increase | 1.00 (0.94, 1.06) | 0.969 |
|  | CD8 | High vs Low | 0.46 (0.10, 2.10) | 0.317 |
|  | Race | European American vs African American | 1.64 (0.41, 6.63) | 0.486 |

| Epithelial CD8 Expression |
| --- |

| **Outcome** | **Variable** |  | **HR (95% CI)** | **P-value** |
| --- | --- | --- | --- | --- |
| OS | Age | 1 Year Increase | 1.02 (0.97, 1.07) | 0.412 |
|  | CD8 | High vs Low | 0.70 (0.24, 2.01) | 0.508 |
|  | Race | European American vs African American | 1.31 (0.48, 3.54) | 0.598 |
| DSS | Age | 1 Year Increase | 1.01 (0.95, 1.07) | 0.868 |
|  | CD8 | High vs Low | 0.51 (0.11, 2.30) | 0.382 |
|  | Race | European American vs African American | 2.86 (0.62, 13.25) | 0.180 |
